# Nitrofurantoin-induced liver injury: long-term follow-up in two prospective DILI registries

**DOI:** 10.1007/s00204-022-03419-7

**Published:** 2022-11-22

**Authors:** Fernando Bessone, Antonella Ferrari, Nelia Hernandez, Manuel Mendizabal, Ezequiel Ridruejo, Alina Zerega, Federico Tanno, Maria Virginia Reggiardo, Julio Vorobioff, Hugo Tanno, Marco Arrese, Vinicius Nunes, Martin Tagle, Inmaculada Medina-Caliz, Mercedes Robles-Diaz, Hao Niu, Ismael Alvarez-Alvarez, Camilla Stephens, M. Isabel Lucena, Raul J. Andrade

**Affiliations:** 1 Hospital Provincial del Centenario, Rosario, Argentina; 2 Hospital de Clínicas, Montevideo, Uruguay; 3 Hospital Universitario Austral, Buenos Aires, Argentina; 4 Centro de Educación Médica e Investigaciones Clínicas (CEMIC), Buenos Aires, Argentina; 5 Hospital Allende, Ciudad de Córdoba, Argentina; 6 Pontificia Universidad Católica de Chile, Santiago, Chile; 7 Hospital Universitário Prof. Edgard Santos-UFBA, Salvador, Brazil; 8 Clínica Anglo Americana, Lima, Peru; 9 Servicios de Aparato Digestivo y Farmacología Clínica, Hospital Universitario Virgen de la Victoria, Instituto de Investigación Biomédica de Málaga y Plataforma en Nanomedicina-IBIMA Plataforma BIONAND, Universidad de Málaga, Málaga, Spain; 10 Centro de Investigación Biomédica en Red Enfermedades Hepáticas y Digestivas (CIBERehd), Madrid, Spain

**Keywords:** Hepatotoxicity, Autoimmune-like hepatitis, Nitrofurantoin, Drug-induced liver injury

## Abstract

Nitrofurantoin is a synthetic antibiotic that is recommended as first-choice treatment for uncomplicated urinary tract infections. The prescription of this drug has increased dramatically, especially in Latin American countries. We described the demographics, clinical characteristics, biochemical features, and outcome of nitrofurantoin-induced liver injury. We analyzed 23 cases from the Latin American DILI Network (LATINDILI) and the Spanish DILI Registry. Causality was assessed with the RUCAM and RECAM scale. Of the 23 DILI cases included in our series, 96% patients were women, and the mean age of the whole cohort was 61 years. The median time of drug exposure was 175 days (interquartile range [IQR] 96–760), with 11 patients who were prescribed nitrofurantoin for more than six months. Hepatocellular damage was the most frequent pattern of liver injury (83%), and nearly half of the patients had an asymptomatic presentation (52%). Neither death nor liver transplantation was documented in this series. Overall, 65% of the patients (*n* = 15) presented with positive autoantibody titres. The median time to resolution was 81 days (IQR 57–141), and 15 patients (83%) recovered within six months. Five patients (22%) developed nitrofurantoin-induced autoimmune-like hepatitis (NI-AILH), of whom two were characterized by a persistent increase in transaminases that required immunosuppressive treatment to achieve normalization of liver enzymes. Clinicians who prescribe nitrofurantoin should be aware that patients who had taken nitrofurantoin for a long term may be at risk of developing nitrofurantoin-induced autoimmune-like hepatitis.

**Supplementary Information:**

The online version contains supplementary material available at 10.1007/s00204-022-03419-7.

## Introduction

Nitrofurantoin is a synthetic antibiotic derived from nitrofuran that has been available for treatment of uncomplicated urinary tract infections (UTI) since 1953, and was widely prescribed during the following two decades. In 1970, with the advent of new drugs such as trimethoprim/sulfamethoxazole (TMS) and beta-lactam antibiotics, its use decreased in clinical practice (Huttner et al. 2015). However, the development of resistance to TMS and fluoroquinolones combined with limited development of new oral antibiotics has led to changes in clinical practice guidelines from the main infectious disease societies (Gupta et al. 2011). Hence, nitrofurantoin is again recommended as first-choice treatment for uncomplicated UTI in many clinical guidelines, including Spain, Europe, the United States, and Argentina (Malmros et al. 2019; Kang et al. 2018; Langner et al. 2021; Nemirovsky et al. 2020). This has led to a high rate of nitrofurantoin prescription. However, many clinical guidelines also recommend alternative antibiotics for UTI that could result in differences in prescription rates between physicians and countries. The adverse effects described for use of short periods of treatment are usually mild and reversible, mainly gastrointestinal, such as nausea, abdominal discomfort, and headache (Huttner et al. 2015). However, use during prolonged periods indicated as prophylaxis of recurrent UTI at doses of 50–100 mg/day, or as a postcoital single dose, is common in clinical practice and associated with severe adverse reactions such as pulmonary fibrosis and hepatotoxicity (Ortega Martell et al. 2019). Due to safety concerns, this has led to contraindications of nitrofurantoin as prophylaxis in some European countries.

Prospective DILI registries and nationwide studies have reported a range from 4.2 to 4.7% of liver damage attributed to nitrofurantoin among all the DILI cases (Björnsson et al. 2013, Björnsson et al. 2022; Chalasani et al. 2015). The incidence rate of nitrofurantoin-induced liver injury was reported to be 1 in every 1,369 patients taking nitrofurantoin based on a population study from Iceland (Björnsson et al. 2013). Nitrofurantoin-induced liver injury presents a broad phenotypic spectrum, ranging from transient increases in liver enzymes, icteric hepatitis, acute liver failure and even death, with the most frequent biochemical pattern at presentation being hepatocellular (Björnsson 2017). It may also mimic autoimmune hepatitis (AIH), referred to as drug-induced autoimmune-like hepatitis (DI-AILH), presenting with marked elevations of alanine aminotransferase (ALT), increased gamma globulin levels, and positive anti-nuclear antibody (ANA) and/or anti-smooth muscle antibody (ASMA) titres (Björnsson et al. 2010; Sherigar et al. 2012; Hydes et al. 2014). In a retrospective analysis of DI-AILH cases included in an AIH database, nitrofurantoin together with minocycline accounted for 92% (22/24) of the DI-AILH case series (Björnsson et al. 2010).

The aim of this study was to describe demographics, clinical, and biochemical features, and outcome of drug-induced liver injury (DILI) related to nitrofurantoin in patients enrolled in the Latin American DILI Network (LATINDILI) and the Spanish DILI Registry.

## Materials and methods

This study included all nitrofurantoin-induced liver injury cases that were prospectively enrolled into the LATINDILI (*n* = 20) and the Spanish DILI registry (*n* = 3) from the initiation of these registries (2011 and 1994, respectively) up to 2020. Each case was evaluated by a local physician and then referred to the coordinating center at the University of Málaga where a panel of DILI experts assessed the cases before being included in the database. A structured report form was used to record patient data, including details related to: (I) time between initial medication intake and onset of liver disease and between discontinuation of the suspected agent and improvement or recovery of liver dysfunction; (II) specific serology and biochemistry tests to rule out viral hepatitis, autoimmune and metabolic liver disorders, appropriate imaging tests to exclude biliary obstruction and preexisting liver disease; (III) outcome of liver damage.

Only cases considered drug-related, according to expert clinical judgment, were evaluated using the Roussel Uclaf Causality Assessment Method (RUCAM) and the Revised Electronic Causality Assessment Method (RECAM) (Hayashi et al. 2022). The biochemical criteria for DILI were those published by Aithal et al. (2011). The clinical pattern of liver injury was classified as hepatocellular, mixed or cholestatic according to biochemical parameters and severity was classified as mild, moderate, severe or fatal/liver transplantation according to the DILI severity index scale (Aithal et al. 2011).

Cases with nitrofurantoin-induced autoimmune-like hepatitis (NI-AILH) were diagnosed based on the following criteria: (I) fulfill the biochemical criteria for DILI, after ruling out alternative causes of liver disease; (II) no underlying liver disease before taking the suspected drug; (III) intake of a drug prior to onset of liver damage and, meet at least two of the following items: positive autoantibodies (ANA, ASMA, and anti-liver kidney microsomal type 1 [LKM1]), increase of immunoglobulin G levels above the upper limit of normal (ULN), or liver biopsy suggestive for DI-AILH. Liver biopsy was considered as suggestive for NI-AILH if one of the following features were present: interface hepatitis, portal and periportal lymphoplasmacytic and eosinophilic infiltration and others similar to AIH, with the exception of advanced fibrosis and cirrhosis (Mack et al. 2020).

### Statistical analysis

Demographic and clinical data for subjects included in the study were examined using descriptive statistics. For quantitative data, mean and standard deviation (SD), or median and interquartile range (IQR, i.e., the difference between the 25th and 75th percentiles) were calculated. Qualitative variables were presented using frequency distributions. Differences between groups were assessed with the Student’s *t* test or Mann–Whitney *U* test, as appropriate, while qualitative variables were compared using the *χ*^2^ test or Fisher’s exact test, as appropriate. Percentages were calculated based on available data, and no imputation methods were performed. All statistical analyses were performed using R version 4.1.3 (R Core Team 2022). A two-sided *p* value lower than 0.05 was deemed as statistically significant.

## Results

A total of 23 cases associated with DILI due to nitrofurantoin were analyzed. Among the 20 cases included in the LATINDILI Network, the majority were enrolled in Argentina (*n* = 13, 55%) and the remaining cases were reported from Chile (*n* = 3), Uruguay (*n* = 2), Brazil (*n* = 1), and Peru (*n* = 1).

Of the 23 nitrofurantoin-induced DILI cases included in our series, 22 (96%) were women, and the mean age of the total cohort was 61 years. Application of the RUCAM scale resulted in “Highly probable” for three (13%) cases, “Probable” for 16 (70%) cases, and “Possible” for the four (17%) remaining cases. In addition, when the RECAM was applied, seven cases were “Probable” (30%), and the remaining 16 were “Possible” (70%). The main indication for nitrofurantoin was long-lasting therapy prescribed for prophylaxis of recurrent lower UTI (*n* = 21, 91%), of which 11 (52%) were prescribed nitrofurantoin for more than six months. Only in two patients (8.7%) was nitrofurantoin indicated as first-line treatment prescribed for less than three weeks.

Regarding drug exposure, the median time was 175 days (IQR 96–760), with a median latency of 143 days (IQR 83–757). Of note, three patients (13%), nine patients (39%), and 11 patients (48%) developed hepatotoxicity in < 30 days, within 30–180 days and > 180 days from nitrofurantoin initiation, respectively. Detection of liver injury occurred after nitrofurantoin discontinuation in four cases, the longest time being 21 days after discontinuation. The median nitrofurantoin dose was 100 mg/day (range 50–450 mg/day), with 17 patients (74%) receiving 100 mg/day.

Hepatocellular damage was the most frequent pattern of liver injury in our series (19 patients, 83%), with the remaining cases presenting either mixed or cholestatic injury (two patients each, 8.7%). Demographic characteristics, clinical, and laboratory findings are presented in Table 1.

**Table 1:** Demographic, clinical, and biochemical characteristics of nitrofurantoin-induced liver injury cases

	Nitrofurantoin DILI cases (*n* = 23)
Age (years), mean ± SD	61 ± 14
Female sex, *n* (%)	22 (96)
Body mass index, mean ± SD	25 ± 2.4
Arterial hypertension, *n* (%)	9 (39)
Type of liver injury, *n* (%)	
Hepatocellular	19 (83)
Cholestatic	2 (8.7)
Mixed	2 (8.7)
Jaundice, *n* (%)	11 (48)
Hospitalization, *n* (%)	9 (39)
Fever, *n* (%)	2 (8.7)
Arthralgia, *n* (%)	4 (20)
Duration of therapy (days), median (IQR)	175 (96–760)
Time to DILI recognition (latency), median (IQR)	143 (83–757)
Causality assessment RUCAM, *n* (%)	
Possible	4 (17)
Probable	16 (70)
Highly probable	3 (13)
Causality assessment RECAM, *n* (%)	
Possible	16 (70)
Probable	7 (30)
Liver parameters at DILI recognition (× ULN), median (IQR)	
Total bilirubin	1.5 (1.0–8.1)
AST	11 (5.9–23)
ALT	13 (8.9–21)
ALP	1.4 (1.0–2.0)
Positive autoantibody titres, *n* (%)	15 (65)
ANA	14 (64)
ASMA	4 (18)
LKM-1^a^	1 (8.3)
DI-AILH, *n* (%)	5 (22)
Severity, *n* (%)	
Mild	12 (52)
Moderate	10 (44)
Severe	1 (4.0)
Time to resolution (days), median (IQR)	81 (57–141)

*ALT* alanine aminotransferase, *ALP* alkaline phosphatase, *ANA* anti-nuclear antibodies, *AST* aspartate aminotransferase, *ASMA* anti-smooth muscle antibodies, *DI-AILH* drug-induced autoimmune-like hepatitis, *IQR* interquartile range, *LKM-1* liver kidney microsomal type 1, *RECAM* Revised Electronic Causality Assessment Method, *RUCAM* Roussel Uclaf Causality Assessment Method, *SD* standard deviation, *ULN* upper limit of normal

^a^Data of LKM-1 autoantibodies were available for 12 patients

The most common symptom at presentation was jaundice (48%) followed by arthralgia (20%) and fever (8.7%), and 39% required hospitalization. Fifty-two percent of the patients were asymptomatic and liver damage was detected during routine blood analyses. No patient developed skin rash, asthenia, abdominal pain, or pruritus.

Regarding serology, 65% of the patients (*n* = 15) presented positive autoantibody titers. Fourteen patients (64%) presented ANA, with a minimum titer of 1/20 and a maximum of 1/1280, with 67% of these patients having a titer greater than or equal to 1/320. Eighteen percent (*n* = 4) presented positive ASMA titres, and one patient had positive LKM-1 titres.

The biochemical profile of the cases on admission was characterized by a median elevation in total bilirubin (TBL) of 1.5 × ULN. Serum aspartate aminotransferase (AST) and alanine aminotransferase (ALT) were increased in all patients with a median value of 11 and 13 × ULN, respectively. Median alkaline phosphatase (ALP) elevation was 1.4 × ULN. Severity of the cases was classified as mild in 12 (52%), moderate in ten (44%), and severe in one patient (4%). Neither death nor liver transplantation was documented in this series.

The median time to resolution was 81 days (IQR 57–141). Fifteen patients (83%) presented liver tests normalization within six months, while three patients (17%) required > 6 months for complete liver profile normalization. Resolution time could not be obtained for the remaining five patients due to loss to follow-up. Three patients were lost to follow-up within the first month after diagnosis, one as outpatient and two just after being discharged, all of them with a moderate increase in transaminases. The remaining two patients were lost to follow-up within six months after DILI diagnosis, when they presented a liver profile near to normalization.

When comparing clinical features, biochemical parameters, and severity in patients with normalized liver tests before and after 90 days, no major differences were observed between the two groups. However, a higher level of TBL and an inclination towards higher severity were detected in the group requiring longer time to resolution, although no statistical significance was reached (Table 2).

**Table 2:** Demographic, clinical, and biochemical characteristics of nitrofurantoin-induced liver injury cases according to time to resolution

	Time to resolution < 90 days (*n* = 10)	Time to resolution ≥ 90 days (*n* = 8)	*p* value
Age (years), mean ± SD	65 ± 13	64 ± 13	0.911
Female sex, *n* (%)	10 (100)	8 (100)	1.000
Body mass index, mean ± SD	26 ± 2.7	24 ± 0.7	0.028
Arterial hypertension, *n* (%)	5 (50)	3 (43)	1.000
Type of liver injury, *n* (%)			0.183
Hepatocellular	9 (90)	6 (75)	
Cholestatic	0 (0)	2 (25)	
Mixed	1 (10)	0 (0)	
Jaundice, *n* (%)	3 (30)	6 (75)	0.153
Hospitalization, *n* (%)	3 (30)	4 (50)	0.631
Duration of therapy (days), median (IQR)	281 (125–829)	655 (124–1012)	0.829
Time to DILI recognition (latency), median (IQR)	250 (109–792)	665 (117–990)	0.697
Liver parameters at DILI recognition (× ULN), median (IQR)			
Total bilirubin	1.2 (0.8–1.6)	5.5 (2.4–9.5)	0.043
AST	12 (6.4–16)	13 (8.8–40)	0.408
ALT	14 (8.5–19)	15 (11–33)	0.633
ALP	1.2 (0.9–1.8)	2.2 (1.5–3.8)	0.083
Positive autoantibody titres, *n* (%)	6 (60)	7 (88)	0.314
DI-AILH, *n* (%)	2 (20)	3 (38)	0.608
Severity, *n* (%)			0.100
Mild	7 (70)	2 (25)	
Moderate	3 (30)	5 (63)	
Severe	0 (0)	1 (13)	

*ALT* alanine aminotransferase, *ALP* alkaline phosphatase, *AST* aspartate aminotransferase, *DI-AILH* drug-induced autoimmune-like hepatitis, *IQR* interquartile range, *SD* standard deviation, *ULN* upper limit of normal

**Fig. 1:**
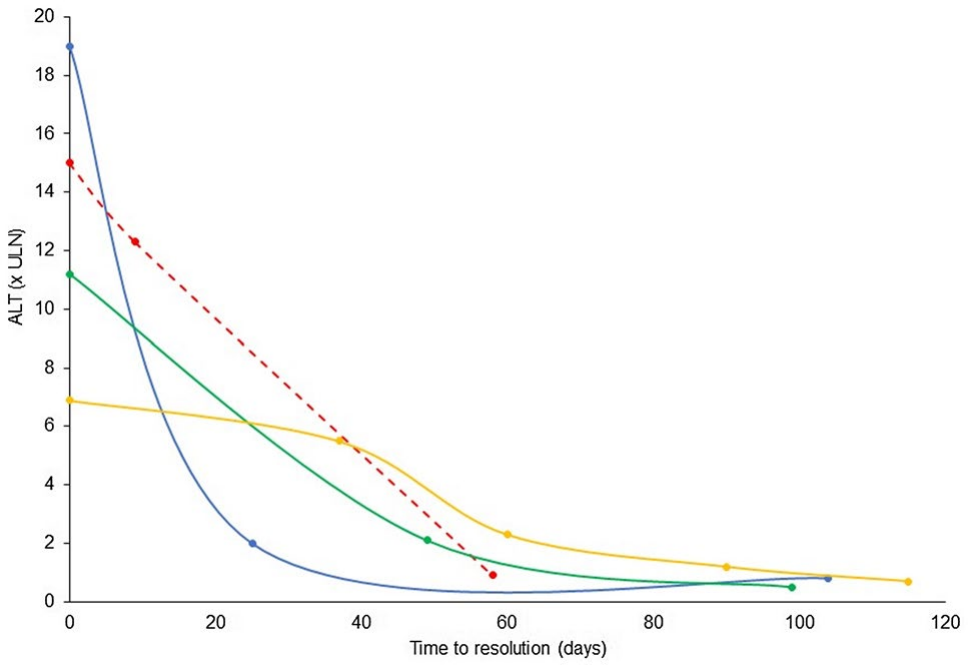
Evolution of alanine aminotransferase (ALT) levels over time until normalization in cases of nitrofurantoin-induced autoimmune-like hepatitis (NI-AILH). Four of five NI-AILH cases had complete follow-up until normalization and are included in the figure. The evolution of ALT in times upper limit of normal (ULN) in one case who received immunosuppressive therapy is represented with a dotted line

Five patients developed NI-AILH, who were all women, four with hepatocellular and one with cholestatic type of liver injury. Two of the patients were characterized by a persistent increase in ALT/AST that required immunosuppressive treatment to achieve normalization of liver tests. One patient was treated with corticosteroid monotherapy, while combined treatment with corticosteroids and azathioprine was prescribed to the other patient. With regard to the latter patient, the liver profile normalized after nine months of combined corticosteroid and azathioprine treatment and both treatments were then stopped. However, the patient had a relapse with elevated liver profile values four years later and was once again restarted on combined corticosteroid and azathioprine treatment. This was the only NI-AILH patient who had a relapse with elevated liver profile values after the reported episode. Evolution of ALT over time until normalization is depicted in Fig. 1. All five patients presented ANA titres ranging from 1/320 to 1/1280. Compared with the 18 DILI cases, NI-AILH cases presented a tendency towards being older (72 years vs. 58 years), although the difference was not statistically significant, most likely due to the limited number of patients (Table 3). Detailed characteristics of the five NI-AILH cases are presented in Supplementary Table 1.

**Table 3:** Demographic, clinical, and biochemical characteristics of nitrofurantoin-induced autoimmune-like hepatitis cases

	DI-AILH (*n* = 5)	DILI (*n* = 18)	*p* value
Age (years), mean ± SD	72 ± 13	58 ± 13	0.049
Female sex, *n* (%)	5 (100)	17 (94)	1.000
Body mass index, mean ± SD	24 ± 1.4	25 ± 2.6	0.282
Arterial hypertension, *n* (%)	4 (80)	5 (29)	0.116
Type of liver injury, *n* (%)			0.654
Hepatocellular	4 (80)	15 (83)	
Cholestatic	1 (20)	1 (6.0)	
Mixed	0 (0)	2 (11)	
Jaundice, *n* (%)	2 (40)	9 (50)	1.000
Hospitalization, *n* (%)	2 (40)	7 (39)	1.000
Duration of therapy (days), median (IQR)	387 (134–1847)	163 (76–751)	0.297
Time to DILI recognition (latency), median (IQR)	357 (135–1472)	137 (76–748)	0.371
Liver parameters at DILI recognition (× ULN), median (IQR)			
Total bilirubin	1.5 (1.4–2.7)	1.5 (0.8–9.2)	0.852
AST	13 (11–16)	9.3 (5.5–26)	0.333
ALT	15 (11–19)	13 (8.2–21)	0.766
ALP	2.6 (1.4–3.7)	1.3 (0.9–1.8)	0.118
Severity, *n* (%)			1.000
Mild	3 (60)	9 (50)	
Moderate	2 (40)	8 (44)	
Severe	0 (0)	1 (6.0)	
Time to resolution (days), median (IQR)	99 (77–104)	60 (45–148)	0.459

*ALT* alanine aminotransferase, *ALP* alkaline phosphatase, *AST* aspartate aminotransferase, *DI-AILH* drug-induced autoimmune-like hepatitis, *IQR* interquartile range, *SD* standard deviation, *ULN* upper limit of normal

An analysis of 21 patients that had taken nitrofurantoin for more than three weeks, comparing 11 patients who took nitrofurantoin for more than six months with ten patients who took it for a shorter period of time, was performed (Table 4). Patients who were exposed for a longer period of time showed a trend towards presenting increased TBL levels (median 6.7 × ULN vs. 1.2 × ULN). Interestingly, patients who were exposed to nitrofurantoin more than six months tended towards higher prevalence of positive autoantibody titres than patients with shorter drug exposure, although the difference did not reach statistical significance (91% vs. 50%, *p* = 0.063). Conversely, there was no association between taking nitrofurantoin for a longer period of time and development of chronic DILI, i.e., no resolution within one year.

**Table 4:** Demographic, clinical, and biochemical characteristics of nitrofurantoin-induced liver injury cases according to duration of therapy

	Duration of therapy < 180 days (*n* = 10)	Duration of therapy ≥ 180 days (*n* = 11)	*p* value
Age (years), mean ± SD	61 ± 13	59 ± 15	0.768
Female sex, *n* (%)	9 (90)	11 (100)	0.476
Body mass index, mean ± SD	26 ± 2.9	24 ± 1.5	0.073
Arterial hypertension, *n* (%)	2 (20)	6 (55)	0.183
Type of liver injury, *n* (%)			0.449
Hepatocellular	7 (70)	10 (91)	
Cholestatic	1 (10)	1 (9.1)	
Mixed	2 (20)	0 (0)	
Jaundice, *n* (%)	3 (30)	7 (64)	0.198
Hospitalization, *n* (%)	3 (30)	4 (36)	1.000
Time to DILI recognition (latency), median (IQR)	103 (63–127)	761 (656–1521)	< 0.001
Liver parameters at DILI recognition (× ULN), median (IQR)			
Total bilirubin	1.2 (1.0–1.6)	6.7 (1.3–9.2)	0.098
AST	11 (5.5–15)	10 (8.5–31)	0.439
ALT	12 (7.1–19)	14 (11–25)	0.398
ALP	1.3 (1.0–2.0)	1.4 (0.9–1.7)	0.944
Positive autoantibody titres, *n* (%)	5 (50)	10 (91)	0.063
DI-AILH, *n* (%)	2 (20)	3 (27)	1.000
Severity, *n* (%)			0.456
Mild	7 (70)	4 (36)	
Moderate	3 (30)	6 (55)	
Severe	0 (0)	1 (9.0)	
Time to resolution (days), median (IQR)	73 (57–97)	99 (59–195)	0.416

*ALT* alanine aminotransferase, *ALP* alkaline phosphatase, *AST* aspartate aminotransferase, *DI-AILH* drug-induced autoimmune-like hepatitis, *IQR* interquartile range, *SD* standard deviation, *ULN* upper limit of normal

Seven patients underwent liver biopsy. The most frequent histological features were interphase hepatitis and lymphocytic infiltrate (in four patients), and an advanced degree of fibrosis (in three patients) (Table 5).

**Table 5:** Histological features of nitrofurantoin-induced liver injury cases

Sex/Age	Clinical presentation/clinical pattern	Histological findings
F/48	Jaundice/hepatocellular	Widened portal spaces with dense lymphocytic and eosinophilic infiltrate. Interphase hepatitis and ductal damage. Lobular necrosis and hepatocanalicular cholestasis. No fibrosis
F/70	Asymptomatic hypertransaminasemia/cholestatic	Interphase hepatitis. Scarce portal plasma cells and marked eosinophilic portal infiltrate. No fibrosis
F/54	Jaundice/hepatocellular	Mild portal inflammatory activity with lymphocytic predominance without lobular changes or interphase hepatitis. No fibrosis
F/86	Jaundice/hepatocellular	Moderate portal inflammatory activity with lymphocytic predominance associated with interphase hepatitis. Fibrotic septa lobulated liver parenchyma forming an incipient cirrhosis
F/28	Jaundice/hepatocellular	Pattern compatible with acute hepatitis associated with portal eosinophilia and periportal ductular proliferation
F/56	Jaundice/hepatocellular	Ductal proliferation and portal infiltrate with lymphocytic predominance. Fibrous septa forming regeneration nodules
F/69	Asymptomatic hypertransaminasemia/hepatocellular	Pattern of chronic liver disease with interphase hepatitis. Tendency to the formation of portal-port bridges with incomplete regeneration nodules

*F* female

## Discussion

Nitrofurantoin is a commonly prescribed antibiotic for treating acute uncomplicated UTI, but also as prophylaxis for recurrent UTI for prolonged periods. In our series of 23 nitrofurantoin-induced liver injury cases, 91% of the patients received long-term treatment (> 3 weeks). This is in line with previous reports of long-term treatments being more likely to produce DILI (Chalasani et al. 2015). In addition, considerably more cases were detected in Latin America than in Spain. Although nitrofurantoin is recommended for acute cystitis in clinical guidelines in many Latin American countries and Spain, alternative antibiotics, such as fosfomycin and cephalexin, are also recommended and could affect nitrofurantoin prescription rate. Hence, we cannot exclude that the higher number of Latin American nitrofurantoin-induced liver injury cases stem from a higher total number of prescriptions due to both population size and prescription rate compared to Spain. Furthermore, in 2016, the Spanish Agency of Medicines and Health Products (AEMPS) restricted the use of nitrofurantoin to curative treatment of acute cystitis for a maximum of seven consecutive days (Agencia Española de Medicamentos y Productos Sanitarios 2016). However, in Latin America, there are still no restrictions for prescribing nitrofurantoin for prolonged periods of time, a situation that could also explain the higher number of cases reported from our LATINDILI network.

The higher prevalence of uncomplicated UTI in females provides an obvious bias towards nitrofurantoin-induced liver injury in females, which reaches 100% in some published case series (Stricker et al. 1988; Chalasani et al. 2015). Furthermore, nitrofurantoin is not usually recommended in men with UTI, due to frequent concomitant prostatic infections. The need to use other antibiotic regimens in these cases is based on that nitrofurantoin does not reach adequate therapeutic concentrations in the prostate (Huttner et al. 2015). We cannot fully exclude the possibility of women having biological differences that make them more susceptible to nitrofurantoin-induced liver injury. However, it seems more likely that the predominance in identified female DILI cases is the result of differences in prescription rates between men and women.

The mean age in our study was 61 years, which is similar to findings in other published studies (Stricker et al. 1988; Chalasani et al. 2015). In a retrospective study evaluating adverse reactions induced by nitrofurantoin in patients over 65 years of age, 25 of 3400 patients (0.7%) experienced hepatic or pulmonary adverse effects, frequently associated with long-term use of this agent (Claussen et al. 2017). These results emphasize that long-term nitrofurantoin treatment appears to be associated with a higher risk factor of DILI than short-term treatment as indicated previously (Westphal et al. 1994). In line with these data, 11 cases (48%) in our analysis were patients older than 60 years of whom four underwent prolonged treatment (≥ 180 days).

Exposure time and latency varied between the cases in our study, but were mainly prolonged with 48% of the cases having an exposure time longer than one year and 43% a latency of more than a year. This is consistent with observations by Björnsson et al. (2010) who found a mean time of exposure of 24 months when analyzing cases of DI-AILH where nitrofurantoin and minocycline were the main culprits. In addition, Chalasani and colleagues from the DILIN (Chalasani et al. 2015) reported several cases linked to latencies beyond one year, while another study showed an exposure time ranging from 30 days to three years, most of them being longer than six months (Sharp et al. 1980). Indeed, most of the cases were scored as “Probable” when the RUCAM was used, while the new RECAM tool categorized the majority of the cases as “Possible”. These differences could be attributed to this prolonged latency in most of the cases, which is penalized. This issue underlines that, despite the strengths of this easy-to-use computerized scale, the heterogeneity of clinical presentations in DILI may require some adjustments to accommodate for specific drugs. Also, DI-AILH cases are underscored due to the presence of autoimmune features. However, RECAM has been adjusted to compensate for these characteristics in nitrofurantoin and minocycline cases.

We observed that 52% of our patients were asymptomatic at presentation with liver profile elevations detected during routine blood analyses. This finding differs from that of Stricker et al. who found that only 11% of cases in their case series were asymptomatic (Stricker et al. 1988). In accordance with our data, however, these authors also found that jaundice (55%) was the most frequent clinical manifestation. A predominance of hepatocellular type of liver injury, as found in our study (83%), has also been documented by others (Sharp et al. 1980; Stricker et al. 1988; Chalasani et al. 2015). Another frequent feature of DILI induced by nitrofurantoin is positive autoantibodies (Sharp et al. 1980; Stricker et al. 1988). Antinuclear antibodies were present in 64% of our cases, of which 29% also presented positive ASMA titres. A review of reported nitrofurantoin-induced liver injury cases presented even higher percentages of ANA (82%) and ASMA (73%) (Stricker et al. 1988), while another study detected positive ANA and ASMA titres in 78% and 72% of cases, respectively (Sherigar et al. 2012). Most of the cases in our study were mild to moderate, with neither deaths nor liver transplantations. These data have also been found by others showing that nitrofurantoin-induced liver injury is rarely associated with severe liver injury and cirrhosis (Björnsson et al. 2010; de Boer et al. 2017).

We found that the time to recovery varied between the cases in the current study, with 44% requiring more than 90 days to reach complete liver profile normalization. Notably, three cases did not recover within the first six months (17%). Extended time to recovery has also been reported in earlier studies with 24% of nitrofurantoin cases in the DILIN registry presenting elevated liver profiles after 180 days (Chalasani et al. 2015). These cases had more severe injury, while most of our cases experienced mild to moderate liver injury, which might explain the shorter time until full recovery. Indeed, we did not find any major differences in the current study population when stratifying the patients into those with a resolution time shorter and longer than 90 days. Nonetheless, we noted a tendency in higher bilirubin level at detection and severity in the group with longer resolution time, which is consistent with that these patients may have had more extended liver injury and therefore required longer time to resolution. In addition, this group also included cholestatic cases, with ALP elevations often decreasing less rapidly than ALT elevations. Interestingly, four of the six patients with corticosteroid treatment attended follow-up sessions until liver profile normalization, and only one of these patients had a time to resolution shorter than 90 days. These differences could to some extent be the result of different treatment regimes, but also highlights the fact that further studies are required to determine when and how corticosteroid treatment should be used in the context of DILI.

Five cases in our series were identified as NI-AILH after prospective evaluation by an expert committee. Nitrofurantoin is a well-recognized culprit responsible for causing DI-AILH in case reports and series (Appleyard et al. 2010; Björnsson et al. 2010; Sherigar et al. 2012; Hydes et al. 2014). Our NI-AIHL cases showed similar characteristics to 42 nitrofurantoin-induced liver injury with an autoimmune phenotype in the US DILIN registry, except for that our cases progressed with less severe damage and no fatalities were reported (de Boer et al. 2017). The divergence in diagnostic criteria of DI-AILH, which lacks a consensus definition despite recent efforts, could explain the differences. In the latter study, nearly half of the cases were treated with corticosteroids (de Boer et al. 2017). Similarly, two out of five NI-AILH cases in our cohort were under immunosuppressive therapy. Despite that corticosteroid use to treat DILI relies on empirical clinical decisions, its use in this autoimmune phenotype was considered in clinical practice guidelines of DILI (Andrade et al. 2019; Chalasani et al. 2021). Interestingly, one of these cases showed a rapid improvement of the condition, while the other one had a relapse four years later without reexposure and was restarted on long-term combined corticosteroid and azathioprine treatment. This latter case was the only NI-AILH patient with a chronic “self-perpetuating” phenotype, which is, indeed, indistinguishable of idiopathic AIH.

The time from initiating nitrofurantoin treatment to DILI detection varied considerably between the cases in our case series. However, we found few differences in demographic, clinical, and biochemical characteristics when comparing patients with duration of nitrofurantoin therapy shorter and longer than 180 days. We noted a tendency towards higher severity and increased TBL in the group with longer duration of therapy. In fact, extended nitrofurantoin therapy has been associated with increased risk of severe adverse reactions (primarily pulmonary and hepatic events) in a meta-analysis (Muller et al. 2017). Interestingly, a larger proportion of patients with autoantibody presentation had been exposed to nitrofurantoin for more than 180 days. One might hypothesize that longer nitrofurantoin exposure triggers a different liver injury mechanism. Histological and genetic characteristics, not evaluated in our study, could provide further valuable information on such potential mechanistic differences. Longer exposure time could therefore be linked to a higher risk of developing autoimmune liver disease induced by nitrofurantoin. This is in line with that the five NI-AILH cases, all with positive ANA titres, had a notably higher duration of nitrofurantoin treatment.

 While providing important information based on well-vetted cases, our study also has limitations. Some cases were lost to follow-up with the result of limited disease progression details. Furthermore, all decisions about patient management, including immunosuppressive therapy, were made by the physicians in charge with the result of potential differences in treatment regimes.

In conclusion, our study reinforces the concept that nitrofurantoin can induce liver injury, frequently associated with a hepatocellular pattern and an asymptomatic presentation. Autoimmune-like hepatitis needing immunosuppressive therapy is a common phenotype in clinical practice, sometimes indistinguishable from classic AIH. Clinicians who prescribe nitrofurantoin should bear in mind that prolonged therapy could be associated with a major possibility of developing NI-AILH.

## Supplementary Information

Below is the link to the electronic supplementary material.

Supplementary file1 (DOCX 21 KB)

## Data Availability

Data analyzed in this article were obtained from the LATINDILI and the Spanish DILI Registry databases. The databases are not publicly available.
