# Supplementary material for: Nitrofurantoin-induced liver injury: long-term follow-up in two prospective DILI registries
Source: Arch Toxicol. 2022 Nov 22;97(2):593–602. doi: 10.1007/s00204-022-03419-7 (PMC9859893; doi:10.1007/s00204-022-03419-7)
Supplement: Supplementary file 1 — Supplementary file1 (DOCX 21 KB) [file 204_2022_3419_MOESM1_ESM.docx]

**Nitrofurantoin-induced liver injury: Long-term follow-up in two prospective DILI registries**

Bessone F^1^, Ferrari A^1^, Hernandez N^2^. Mendizabal M^3^, Ridruejo E^4^, Zerega A^5^, Tanno F^1^, Reggiardo MV^1^, Vorobioff J^1^, Tanno H^1^, Arrese M^6^, Nunes V^7^, Tagle M^8^, Medina- Caliz I^9^, Robles-Diaz M^9,10^, Niu H^9,10^, Alvarez-Alvarez I^9,10^, Stephens C^9,10^, Lucena MI^9,10^, Andrade RJ^9,10^.

**Affiliations:**

^1^Hospital Provincial del Centenario, Rosario, Argentina.

^2^Hospital de Clínicas, Montevideo, Uruguay.

^3^Hospital Universitario Austral, Argentina.

^4^Centro de Educación Médica e Investigaciones Clínicas (CEMIC), Buenos Aires, Argentina. ^5^Hospital Allende, Ciudad de Córdoba, Argentina.

^6^Pontificia Universidad Católica de Chile, Chile.

^7^Hospital Universitário Prof. Edgard Santos-UFBA, Salvador, Brazil.

^8^Clínica Anglo Americana, Lima, Perú.

^9^Servicios de Aparato Digestivo y Farmacología Clínica, Hospital Universitario Virgen de la Victoria, Instituto de Investigación Biomédica de Málaga y Plataforma en Nanomedicina-IBIMA Plataforma BIONAND, Universidad de Málaga, Málaga, Spain.

^10^Centro de Investigación Biomédica en Red: Enfermedades Hepáticas y Digestivas (CIBERehd), Madrid, Spain.

**Corresponding authors:**

Prof. M. Isabel Lucena

Department of Clinical Pharmacology, Facultad de Medicina, Universidad de Málaga

Boulevard Louis Pasteur, 32

29010 Malaga, Spain

Prof. Fernando Bessone

Department of Gastroenterology and Hepatology

Hospital Provincial del Centenario, University of Rosario School of Medicine

Urquiza 3101,

2000 Rosario, Argentina

**Supplemental Table 1.** Clinical characteristics of nitrofurantoin-induced autoimmune-like hepatitis cases.

| **Age / sex** | **Drug** | **Presentation** | **Hospitalization** | **Latency (months)** | **ANA**  **titres** | **SMA**  **titres** | **IgG**  **(g/L)** | **Biopsy** | **Corticosteroid therapy** | **Time to resolution (days)** |
| --- | --- | --- | --- | --- | --- | --- | --- | --- | --- | --- |
| 86 / F | Nitrofurantoin | Jaundice | Yes | 2 | Positive^*^ | Negative | NA | Yes (suggestive) | No | 104 |
| 66 / F | Nitrofurantoin | Hypertransaminemia | No | 61.5 | 1:160 | Negative | 18.8 | No | Yes | 58 |
| 55 / F | Nitrofurantoin | Jaundice | Yes | 90 | 1:320 | 1:640 | 13.7 | Yes (possible) | Yes | 478 |
| 70 / F | Nitrofurantoin | Hypertransaminemia | No | 4.5 | 1:320 | Negative | NA | Yes (suggestive) | No | 99 |
| 82 / F | Nitrofurantoin | Hypertransaminemia | No | 11.6 | 1:1,280 | Negative | 18.8 | No | No | 77 |

Biopsy findings based on the article by Mack *et al.* (2020). Suggestive: interface hepatitis, portal and periportal lymphoplasmacytic and eosinophilic infiltration, lobular hepatitis, and other features commonly associated with autoimmune hepatitis, with the exception of advanced fibrosis and cirrhosis. Possible: absence of enough details to be considered “suggestive”.

^*^ Titres values not available.

F: female sex; ANA: antinuclear antibodies; SMA: smooth muscle antibodies; IgG: immunoglobulin G; NA: data not available.

Reference: Mack CL, Adams D, Assis DN, Kerkar N, Manns MP, Mayo MJ, et al. (2020) Diagnosis and management of autoimmune hepatitis in adults and children: 2019 practice guidance and guidelines from the American Association for the Study of Liver Diseases. Hepatology 72:671-722. <https://doi.org/10.1002/hep.31065>
